# KeyLoop retractor for global gasless laparoscopy: evaluation of safety and feasibility in a porcine model

**DOI:** 10.1007/s00464-023-10054-5

**Published:** 2023-04-19

**Authors:** Siddhesh Zadey, Harold Leraas, Aryaman Gupta, Arushi Biswas, Pierce Hollier, Joao Ricardo Nickenig Vissoci, Julius Mugaga, Robert T. Ssekitoleko, Jeffrey I. Everitt, Amos H. P. Loh, York Tien Lee, Ann Saterbak, Jenna L. Mueller, Tamara N. Fitzgerald

**Affiliations:** 1 Department of Surgery, Duke University School of Medicine, Durham, NC USA; 2 Association for Socially Applicable Research (ASAR), Pune, MH India; 3 Department of Biomedical Engineering, Pratt School of Engineering, Duke University, Durham, NC USA; 4 Duke Global Health Institute, Durham, NC USA; 5 Trinity College, Duke University, Durham, NC USA; 6 Makerere University College of Health Sciences, Kampala, Uganda; 7 Department of Pathology, Duke University of School of Medicine, Durham, NC USA; 8 Duke-NUS Medical School, SingHealth Duke-NUS Global Health Institute, Singapore, Singapore; 9 Department of Paediatric Surgery, KK Women’s and Children’s Hospital, Singapore, Singapore; 10 Fischell Department of Bioengineering, University of Maryland, College Park, MD USA; 11 Marlene and Stewart Greenebaum Cancer Center, University of Maryland School of Medicine, Baltimore, MD USA

**Keywords:** Gasless laparoscopy, Global surgery, Frugal innovation, Global engineering

## Abstract

**Background:**

Many surgeons in low- and middle-income countries have described performing surgery using gasless (lift) laparoscopy due to inaccessibility of carbon dioxide and reliable electricity, but the safety and feasibility of the technique has not been well documented. We describe preclinical testing of the in vivo safety and utility of KeyLoop, a laparoscopic retractor system to enable gasless laparoscopy.

**Methods:**

Experienced laparoscopic surgeons completed a series of four laparoscopic tasks in a porcine model: laparoscopic exposure, small bowel resection, intracorporeal suturing with knot tying, and cholecystectomy. For each participating surgeon, the four tasks were completed in a practice animal using KeyLoop. Surgeons then completed these tasks using standard-of-care (SOC) gas laparoscopy and KeyLoop in block randomized order to minimize learning curve effect. Vital signs, task completion time, blood loss and surgical complications were compared between SOC and KeyLoop using paired nonparametric tests. Surgeons completed a survey on use of KeyLoop compared to gas laparoscopy. Abdominal wall tissue was evaluated for injury by a blinded pathologist.

**Results:**

Five surgeons performed 60 tasks in 15 pigs. There were no significant differences in times to complete the tasks between KeyLoop and SOC. For all tasks, there was a learning curve with task completion times related to learning the porcine model. There were no significant differences in blood loss, vital signs or surgical complications between KeyLoop and SOC. Eleven surgeons from the United States and Singapore felt that KeyLoop could be used to safely perform several common surgical procedures. No abdominal wall tissue injury was observed for either KeyLoop or SOC.

**Conclusions:**

Procedure times, blood loss, abdominal wall tissue injury and surgical complications were similar between KeyLoop and SOC gas laparoscopy for basic surgical procedures. This data supports KeyLoop as a useful tool to increase access to laparoscopy in low- and middle-income countries.

**Supplementary Information:**

The online version contains supplementary material available at 10.1007/s00464-023-10054-5.

Globally, over 14 million laparoscopic surgeries are performed annually [1]. Laparoscopy has become the standard of care for many operative procedures in high-income countries (HIC), demonstrating lower rates of infection, fewer postoperative complications, and shorter hospital stays compared to open surgery [2–5]. Unfortunately, laparoscopy remains largely inaccessible in low- and middle-income countries (LMICs) due to high cost of equipment and limited availability of medical-grade carbon dioxide, consumable supplies and stable electricity [6]. When laparoscopy is available in LMICs, surgeons are using equipment that was designed for HICs. This equipment is based on fiber optics, which are fragile, and includes multiple parts that must be individually sterilized and can become lost or broken, rendering the entire system unusable. HICs depend on expensive maintenance contracts to maintain this equipment, which are not sustainable for LMICs.

Access to laparoscopic surgery in LMICs could save lives with spillover societal benefits, such as avoidance of stigma associated with certain surgical conditions, earlier return to work and mitigation of catastrophic health expenditure [7, 8]. Many surgeons in LMICs have previous training in laparoscopy and desire to perform laparoscopic cases [9]. They also believe that low-cost and durable laparoscopic systems would benefit their patients. However, scale-up of laparoscopic surgery in these settings will require innovation to address the needs of patients and surgeons.

Limited studies have suggested that gasless laparoscopy can be safe, clinically effective and cost-effective compared to gas-based laparoscopy [10–12]. Innovation in gasless laparoscopy is being explored as a means to improve surgical access in LMICs [12]. General surgeons and gynecologists in LMICs have described performing basic procedures such as exploratory laparoscopy, cholecystectomies and appendectomies using lift devices. Previous devices to facilitate gasless laparoscopy include retractor systems [12–14], abdominal wall-lifting systems [15, 16], inflatable devices [17], and rope-lifting techniques [18]. Most of these studies are case reports in human patients. Hence, there remains limited translational evidence on the efficacy and safety of gasless laparoscopic systems and their feasibility of use.

Adopting a human-centered design approach, we have developed a low-cost, durable, and easily-sterilizable retractor for gasless laparoscopy [19]. Human-centered design is an iterative process whereby intended users of a new technology are interviewed to determine their needs and resources, and how they intend to use the technology [20]. Once a prototype has been developed, the intended users provide feedback regarding the design, such that the device is improved over subsequent iterations. KeyLoop was designed in partnership with our multidisciplinary team to address the needs of patients and surgeons in sub-Saharan Africa [21]. Additionally, we have also developed a low-cost, single-unit, portable laparoscope called KeyScope. KeyScope mitigates fragility by using light-emitting diodes and a color-complementary metal oxide semiconductor camera instead of fiber optics. It can be connected to a laptop computer for presenting real-time video and does not need constant electricity. It can be easily sterilized by immersion in disinfectant. The device has been previously described elsewhere [22] and porcine testing findings will be published in the near future. The aim of the current study was to assess efficacy, safety, and feasibility of KeyLoop compared to standard of care (SOC) gas laparoscopy in pre-clinical porcine studies.

## Materials and methods

### Porcine model

The porcine study was approved by the Duke University Institutional Animal Care and Use Committee (Protocol A219-19-10) and the SingHealth Duke-NUS Institutional Animal Care and Use Committee (Protocol 2020/SHS/1558). The data collected from participating surgeons was determined exempt from review by the Duke University Health System Institutional Review Board (Protocol 00108981) and the SingHealth Duke-NUS Central Institutional Review Board (Protocol 2020/2793). We used a porcine model for comparison of KeyLoop versus gas laparoscopy. Pig anatomy is similar to humans and pigs are commonly used as models for laparoscopy [23], but there are some differences relevant for surgical tasks. The porcine spleen is very large and can obstruct visualization and movement while operating. The gallbladder is substantially intrahepatic and the dissection plane between the liver bed and gallbladder wall is densely adherent, which increases the likelihood of accidental gallbladder puncture or hepatic bleeding. Conversely, the dissection of the cystic artery and cystic duct is much simpler, owing to the increased length of these structures and absence of fat and inflammation in Calot’s triangle. The porcine umbilicus is not anatomically obvious and often too caudal, and therefore the center of the abdomen was used for gaining laparoscopic exposure.

Animals were anesthetized with isoflurane and monitored for hemodynamic stability, appropriate anesthesia, and analgesia by veterinary staff throughout the procedure. We recorded the weight, abdominal diameter, and vital signs such as respiratory rate, heart rate, and oxygen saturation. Animals were sacrificed in accordance with humane protocols at the conclusion of the experiment.

### Laparoscopic procedures

**Fig. 1:**
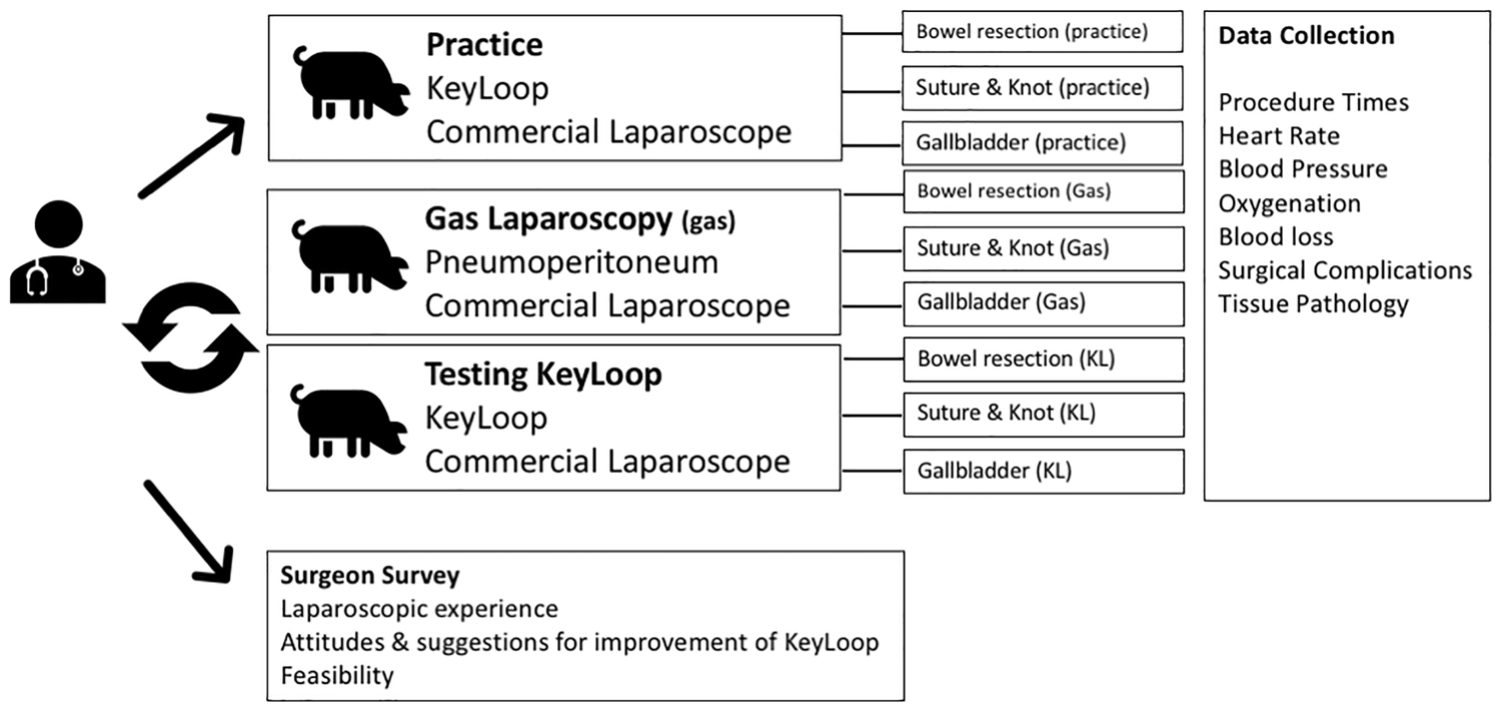
Diagram illustrating the experimental design of porcine experiments. Surgeons completed 4 tasks in all pigs: (1) Achievement of laparoscopic exposure by either KeyLoop or gas laparoscopy, (2) Stapled bowel resection, (3) Gastric suture and intracorporeal knot tie, and (4) Gall bladder removal (Cholecystectomy). The first pig was always a practice pig, and KeyLoop was used. The second and third pigs were block randomized to be either standard of care gas laparoscopy or KeyLoop. At the completion of all surgical tasks, surgeons completed a survey to assess feasibility of the KeyLoop

Surgeons in the United States who routinely perform laparoscopic cases were invited for participation in the study. Figure 1 illustrates the study design. Each surgeon was asked to perform four surgical tasks in each of 3 different animals. The four tasks included: (1) obtaining laparoscopic exposure (either with KeyLoop or gas), (2) stapled small bowel resection, (3) gastric suture with intracorporeal knot tying, and (4) cholecystectomy.

For exposure using gas laparoscopy, surgeons were asked to place a 10 mm laparoscopic port in the center of the abdomen and insufflate with carbon dioxide at a pressure of 15 mmHg [24]. A SOC laparoscope [Karl Storz 5 mm, 0° Endoscope] was inserted through the port and the abdominal cavity was inspected for injuries. For exposure using the KeyLoop, surgeons were asked to make a 10 mm incision in the center of the abdomen, and then introduce their finger into the peritoneum to ensure there were no adhesions between the small bowel and the abdominal wall. The KeyLoop was inserted by introducing the smooth tip and rotating the retractor until the entire loop was under the abdominal wall. The KeyLoop retractor was elevated and secured in place with the bedside stand. A 5 mm port and SOC laparoscope was inserted through the same 10 mm incision as the KeyLoop to assess for any injuries.

For the next three tasks (small bowel resection, gastric suturing with intracorporeal knot tying and cholecystectomy) surgeons were given basic instructions in performing the tasks but were permitted to perform them using techniques that they would use in their standard surgical practice. We asked the surgeons to choose their techniques and perform the procedures the same way in all three animals. For example, surgeons were instructed to find a loop of small bowel and perform a stapled resection of a small segment. The surgeon determined additional laparoscopic port placement, which small bowel loop to choose, with what instrument they would prefer to create a mesenteric window to introduce the stapler and how they wanted to separate the mesentery from the resected segment. For the gastric suturing with intracorporeal knot tying, surgeons could choose port location, the gastric location for the suture, the laparoscopic instruments they would use and the length of the suture they introduced. They were instructed to tie three knots using intracorporeal techniques (no knot pusher). Similarly, for the cholecystectomy, they chose how the assistant surgeon would retract the gallbladder, port placement, laparoscopic instruments and dissection techniques.

For all surgeons, the first pig was used to practice the four tasks using the KeyLoop. This allowed the surgeon to gain familiarity with inserting the KeyLoop and adjust their operative technique to porcine anatomy. It was felt that the surgeons did not need practice with laparoscopic exposure using gas, as all surgeons routinely perform laparoscopic cases in their clinical practice and are familiar with port placement and insufflation. The next two animals were block randomized to either KeyLoop or gas laparoscopy to minimize bias from learning effects. This block randomization ensured that some surgeons performed the tasks with KeyLoop first, and other surgeons performed the tasks with gas laparoscopy first.

Two researchers were present for all experiments. One researcher (who is also a surgeon) served as the operative assistant. The second researcher observed the procedures to record time taken to complete each task and noted any surgical complications or injuries. Vital signs such as heart rate, systolic and diastolic blood pressures, and blood oxygenation level were monitored and recorded. After each task, any blood loss was measured by suctioning into a Lukens trap. No irrigation fluid was used.

### Pathology assessment

Since the KeyLoop exerts sustained pressure on the abdominal wall, histologic evaluation was performed to assess for tissue injury. At the conclusion of the operation, the abdominal wall was marked where the tip of the KeyLoop had been in contact, since that is the point of greatest pressure. This area was sharply removed such that the specimen contained all layers of the abdominal wall from the peritoneum to the skin. When gas laparoscopy had been performed, an anatomically similar area of the abdominal wall was chosen, and a specimen was taken for comparison. The specimens were immersed in formaldehyde for delivery to the histology laboratory. Tissue was sectioned and prepared with standard hematoxylin and eosin staining. The slides were labelled with a coding scheme that allowed the pathologist to be blinded to the experimental conditions. The pathologist is trained in veterinary sciences and has extensive experience with porcine tissues. For each specimen, the pathologist was asked to assess for edema, inflammation, hemorrhage or necrosis and rate each of these categories as not present, mild, moderate or severe. If any tissue damage was found, then the area of damage was recorded in millimeters.

### Surgeons’ feedback survey

After completing the four surgical tasks in all three animals, surgeons in the United States were asked to complete a survey in REDCap [25]. Implied consent was obtained at the start of the survey. The questionnaire is presented in the appendix and included 18 questions investigating the surgeons’ laparoscopic experience, operative experience in LMICs, preference for potentially using the KeyLoop retractor, feasibility of using KeyLoop and suggestions for improvement. Question formats included multiple choice, scoring slider, and open-ended text. Six surgeons from Singapore also performed porcine bowel resections, intracorporeal gastric suturing with knot tying and cholecystectomies in order to provide feedback on the KeyLoop, and these surgeons also completed the survey. Due to differences in funding and administration, the surgeons in Singapore did not have access to the same numbers of pigs and time in the vivarium. Therefore, they completed fewer iterations of the laparoscopic tasks, and the tasks were not rigorously measured, but they were able to provide their opinions regarding the laparoscopic equipment via the REDCap survey.

**Table 1:** Pig characteristics

	Practice (*n* = 5)	KeyLoop (*n* = 5)	Gas (*n* = 5)	*p* values
	Median (IQR)	Median (IQR)	Median (IQR)	
Body weight (kg)	30 (2.2)	29.4 (0.6)	28.8 (0.1)	0.878
Abdominal diameter (cm)	26 (1.5)	25.5 (1.2)	26 (0)	0.885

### Data analysis

For the porcine experiments, we report median values with associated interquartile ranges (IQR) for continuous variables such as times, blood pressures, blood loss, and heart rate. We assessed task-wise learning curves across the animals. We compared the body weights and abdominal diameters across three groups: practice, KeyLoop and gas laparoscopy pigs using the Kruskal–Wallis Test at 1% alpha threshold for statistical significance. Comparison of outcomes (surgical task completion times for efficacy and blood loss for safety) along with vital signs between non-practice KeyLoop and gas experimental conditions was conducted using paired non-parametric Wilcoxon signed-rank statistical tests with a 1% alpha threshold. We used descriptive statistics (proportions and medians with quantiles) and visualizations such as box plots, bar charts, and likert scale for analyzing surgeons’ survey responses. Descriptive statistics were used to analyze pathology findings for injury/complication descriptions.

## Results

Five U.S. surgeons completed a total of 60 laparoscopic tasks in 15 pigs. Of the participating surgeons, one was female and four were male. All surgeons were fellowship-trained and specialty-certified; there were three gastrointestinal laparoscopic surgeons, one surgical oncologist and one pediatric surgeon. All surgeons routinely performed a variety of laparoscopic cases in their surgical practice. Two of the surgeons had previous experience performing surgery in LMICs.

The median (IQR) abdominal diameters (cm) of practice pigs [26 (1.5), *n* = 5], KeyLoop pigs [25.5 (1.2), *n* = 5] and pigs undergoing gas laparoscopy [26 (0), *n* = 5] were similar (*p* = 0.885). The median (IQR) body weights (kg) of practice pigs [30 (2.2), *n* = 5], KeyLoop pigs [29.4 (0.6), *n* = 5] and pigs undergoing gas laparoscopy [28.8 (0.1), *n* = 5] were also similar (*p* = 0.878). Zoometric measures were comparable for animals across practice and experimental conditions (Table 1).

**Fig. 2:**
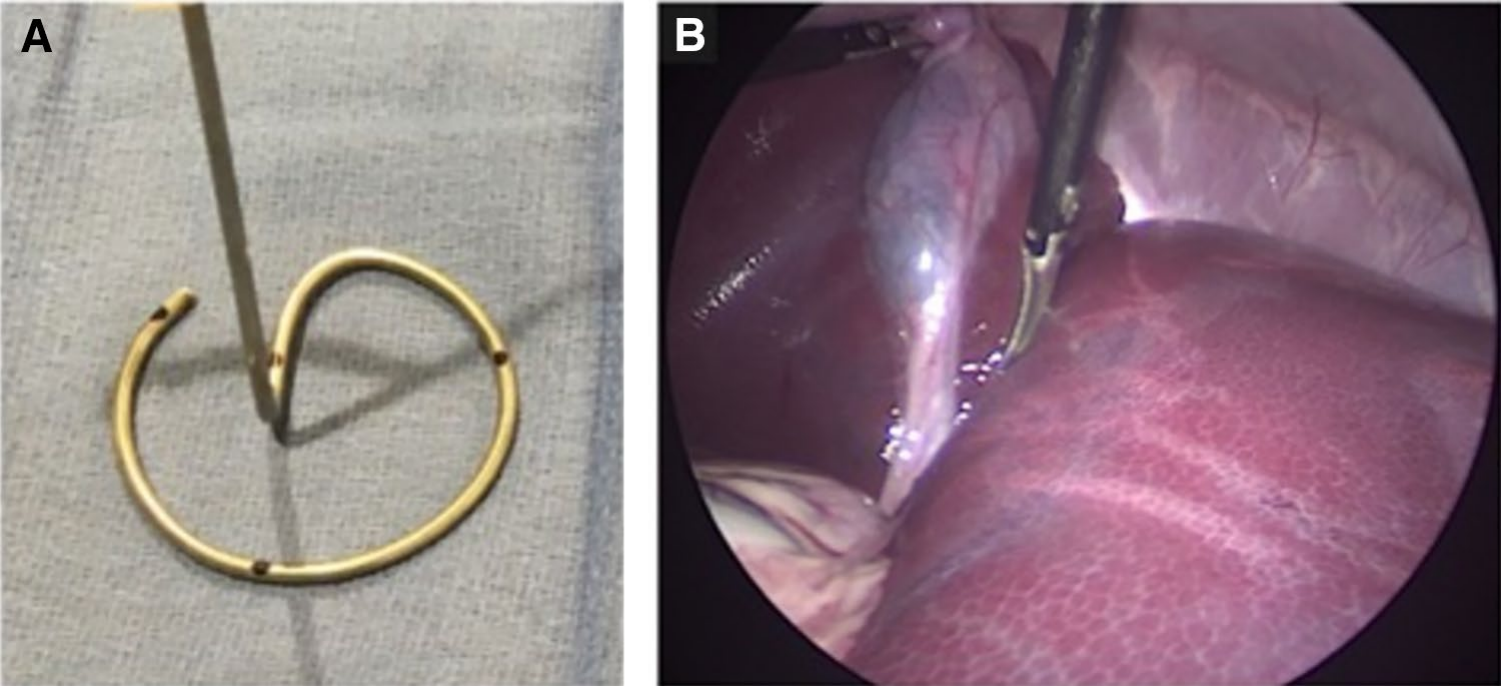
**A** Picture of the KeyLoop used in the experiments. This particular retractor had a 6 cm radius. KeyLoop is semicircular, biocompatible, and made of stain-less steel. The KeyLoop was paired with a custom bedside stand. **B** Intra-abdominal exposure during porcine cholecystectomy. Exposure has been achieved with the KeyLoop (no carbon dioxide was used) and the photograph was taken with the standard of care laparoscope. The gallbladder has been grasped and retracted superiorly. The porcine liver is large, the gallbladder is intrahepatic, and the course of the cystic duct and cystic artery are seen in this view

**Fig. 3:**
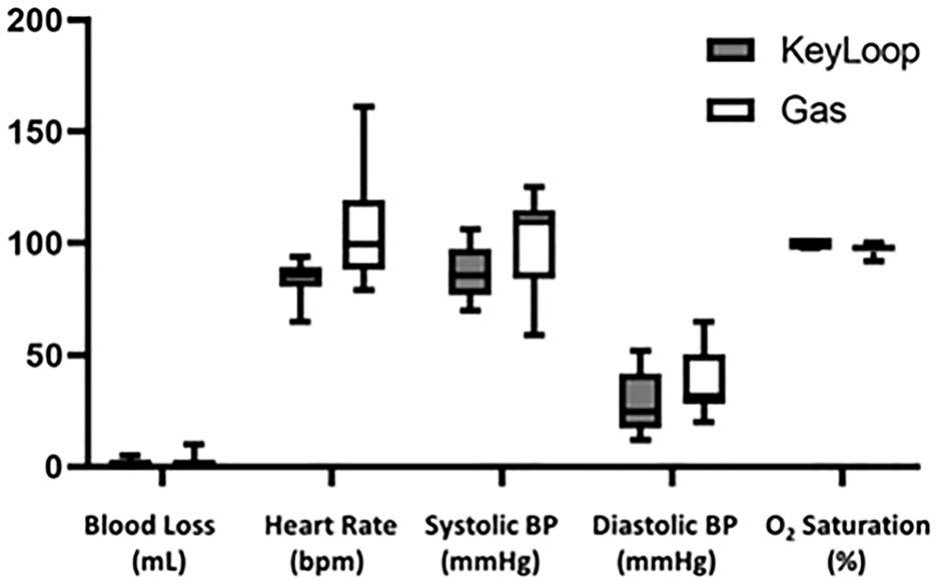
Blood loss and vital signs using KeyLoop versus gas laparoscopy. The filled boxes depict KeyLoop while the unfilled boxes depict gas laparoscopy. The boxes denote interquartile range where the line within shows median. The bars extending from the boxes show minimum and maximum values. In general, blood loss was minimal for all procedures. Blood pressure and heart rate tended to be higher with gas laparoscopy, but this result was not statistically significant (*p* > 0.1). Oxygenation was similar between exposure methods. For blood pressure comparisons, *n* = 8 (KeyLoop: 4, gas: 4)

Figure 2A shows the KeyLoop retractor used for the experiments. Figure 2B shows an intraoperative photograph taken with the standard of care laparoscope during a cholecystectomy using KeyLoop. The surgical exposure was sufficient to safely and effectively perform the required procedures as determined by experienced surgeons. In the photograph, the gallbladder has been grasped and is retracted superiorly. The reader can appreciate that the porcine liver is generous, the gallbladder is intrahepatic, and delineation of the cystic duct and artery are straightforward. In general, blood loss was minimal for all procedures performed (Fig. 3), and there was no significant difference between KeyLoop and gas laparoscopy (*p* > 0.1). Heart rate, systolic blood pressure and diastolic blood pressure tended to be higher during gas laparoscopy, but this result was not statistically significant (*p* > 0.1 for all comparisons). Oxygen saturation was similar between KeyLoop and gas laparoscopy (*p* > 0.1).

Four surgeons punctured the gallbladder in all three animals during the course of removing the gallbladder from the liver bed. When the gallbladder was punctured, there was always adequate surgical exposure, and the puncture was related to the lack of inflammation and a distinct surgical plane between the liver and gallbladder. One surgeon tended to remove the gallbladder utilizing more blunt dissection and less cautery, and this surgeon did not puncture the gallbladder in any of the three experimental conditions. Therefore, there was no difference between KeyLoop and gas laparoscopy in terms of removing the gallbladder intact. One surgeon inserted the KeyLoop too quickly at a sharp angle and caused a bowel injury. There were no bowel injuries due to laparoscopic port placement.

### Operative time

**Fig. 4:**
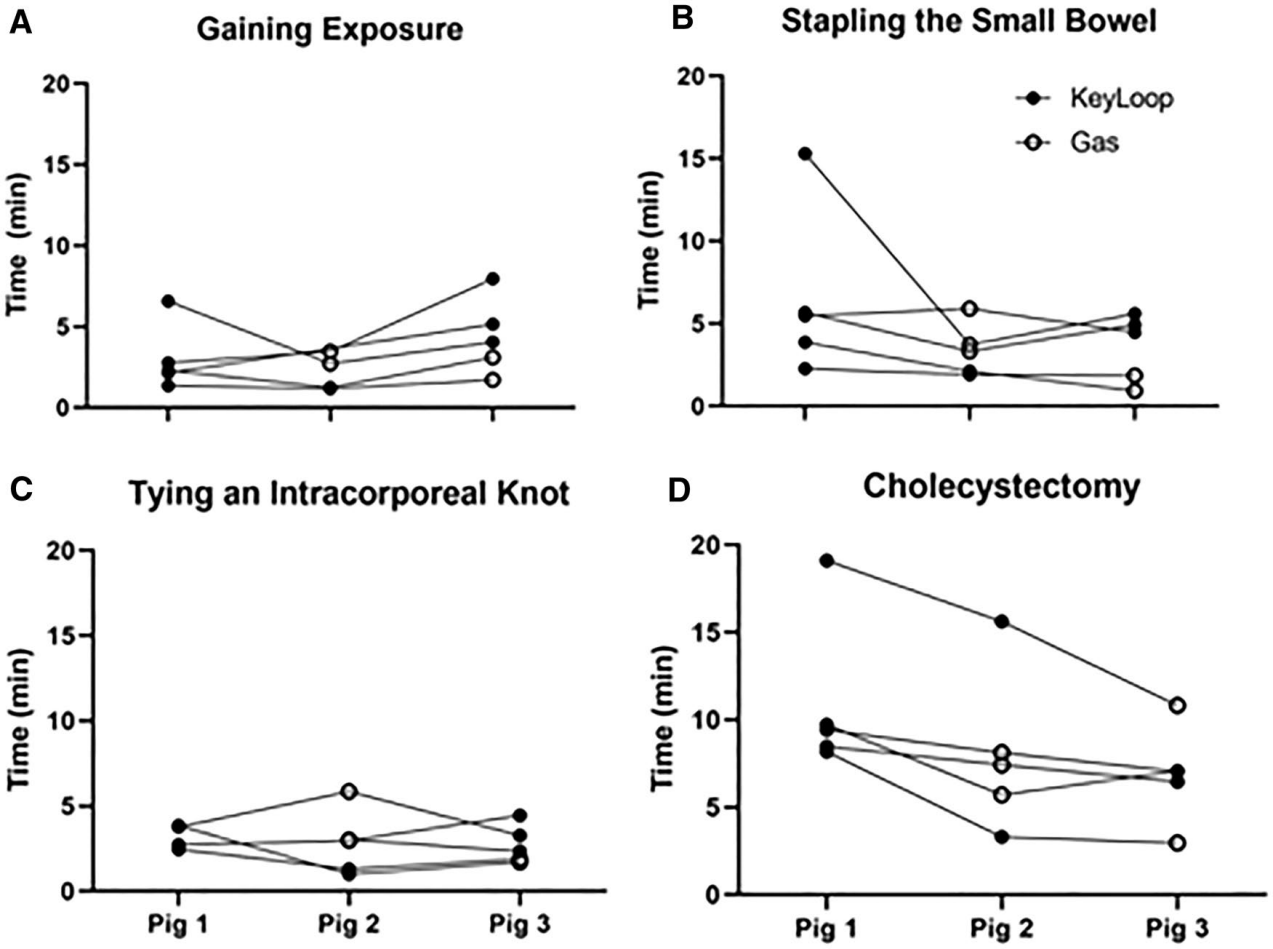
Operative times versus porcine experimental order. Each of the four tasks are shown: **A** Gaining laparoscopic exposure with either KeyLoop or gas laparoscopy. **B** Stapling the small bowel. **C** Gastric suturing and tying 3 intracorporeal knots. **D** Cholecystectomy. Pig 1 was always the practice pig and used KeyLoop. Pigs 2 and 3 were randomized to either KeyLoop (shown as filled circles) or gas laparoscopy (shown as open circles). Individual surgeons are represented by the line connecting their procedures. For stapling the small bowel and performing the cholecystectomy, there appears to be a learning curve, in which procedure times decreased and were more related to the order of the procedure, rather than the method of peritoneal exposure

In general, operative times were similar between KeyLoop and gas laparoscopy and were more affected by a learning curve from surgeons iteratively improving their technique in adaptation to porcine anatomy. This can be seen in Fig. 4, where operative time for each of the four tasks are shown as a function of experimental order. Pig 1 was always the practice pig using KeyLoop. Pigs 2 and 3 were randomized to either KeyLoop (shown as filled circles) or gas laparoscopy (shown as open circles). Individual surgeons are represented by the line connecting their procedures. For more challenging procedures like stapling the small bowel and performing the cholecystectomy, there appears to be a learning curve, in which procedure times decreased and were more related to the order of the procedure, rather than the method of peritoneal exposure.

**Fig. 5:**
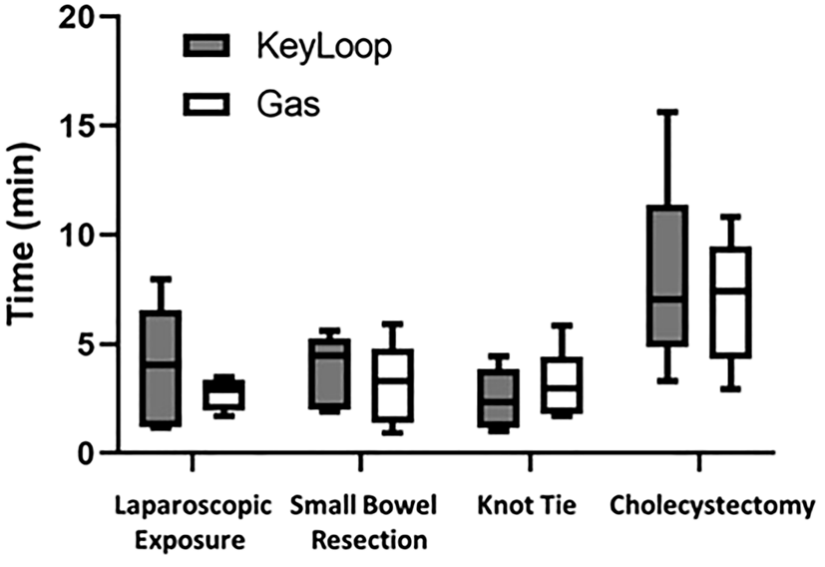
Operative times for the laparoscopic tasks comparing KeyLoop versus gas laparoscopy. The filled boxes depict KeyLoop while the unfilled boxes depict gas laparoscopy. The boxes denote interquartile range where the line within shows median. The bars extending from the boxes show minimum and maximum values. Operative times were not significantly different for any of the tasks

Figure 5 shows the same data, in a different format, in which operative times are directly compared when using KeyLoop versus gas laparoscopy. There were no significant differences in operative times for gaining exposure (KeyLoop [median (IQR)]: 4.1 (3.9); gas: 2.9 (0.7); *p* = 1.0) or performing small bowel resections (KeyLoop: 4.5 (2.8); gas: 3.3 (1.9); *p* = 0.790), gastric suturing with intracorporeal knot tie (KeyLoop: 2.4 (2.0); gas: 3.0 (1.1); *p* = 0.790) or cholecystectomies (KeyLoop: 7.1 (0.6); gas: 7.4 (2.4); *p* = 1.0). Operative times for gaining exposure and tying intracorporeal knots were all less than 10 min, while operative times for more complicated tasks such as small bowel resections and cholecystectomies were all less than 20 min, and most less than 15 min.

### Abdominal wall pathology

The specimens were noted to include the full range of what is expected from the layers of the abdominal wall; skin, skeletal muscle, subcutis, subcutaneous tissue, adipose tissue, and occasionally mammary tissue was noted across specimens. There was no edema, inflammation, hemorrhage or necrosis observed in either the KeyLoop or gas laparoscopy specimens.

### Surgeon assessment of KeyLoop feasibility

**Fig. 6:**
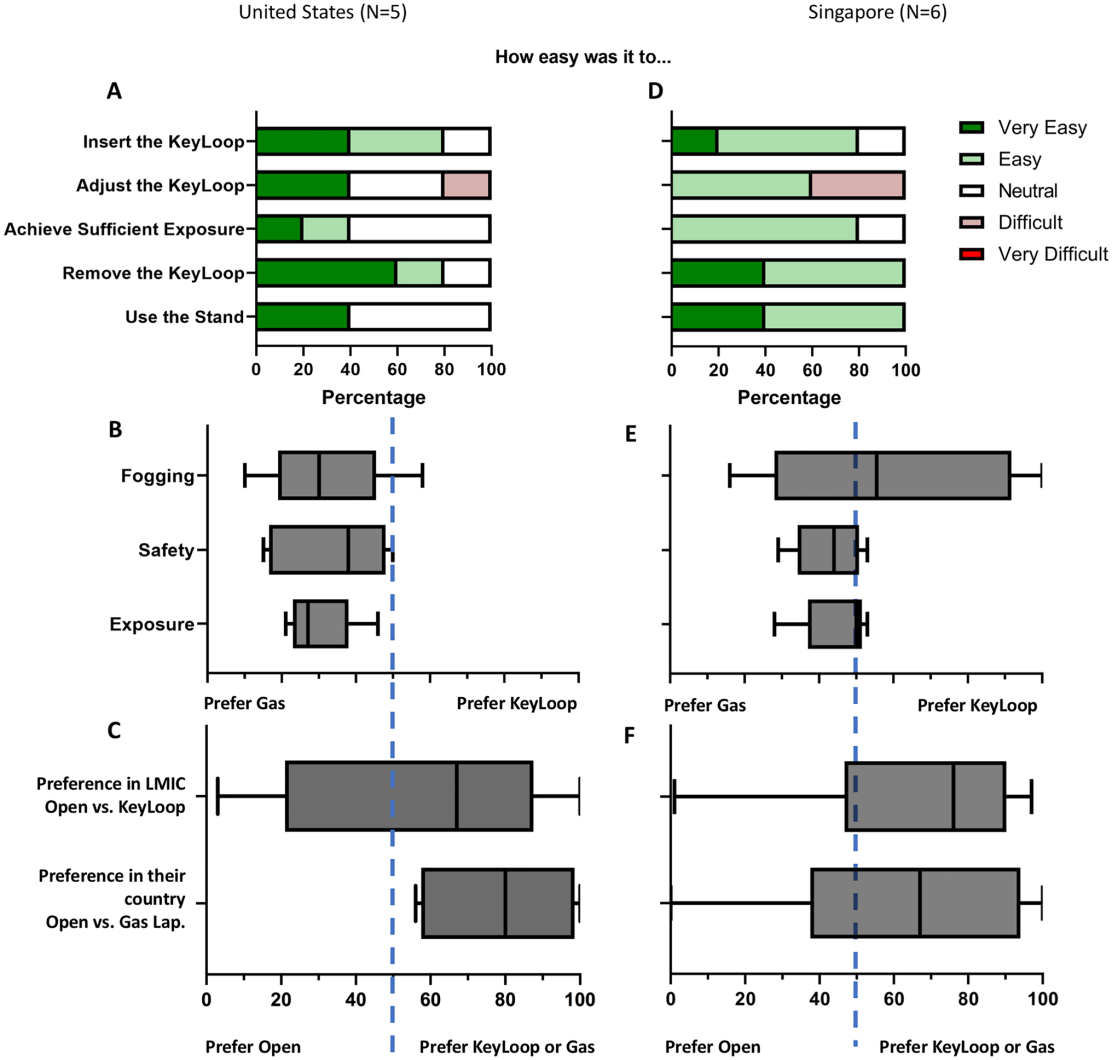
Results of surgeon feedback survey in the U.S. and Singapore. **A** U.S. surgeons found it easy to insert the KeyLoop, achieve sufficient exposure, remove the KeyLoop and adjust the stand. Some surgeons found it more difficult to adjust the KeyLoop. **B** In terms of laparoscope fogging, safety, and peritoneal exposure, the U.S. surgeons preferred to use gas laparoscopy, rather than the KeyLoop. The blue dashed line indicates equal preference. **C** If surgeons were in an LMIC, they had varied responses over whether they would prefer KeyLoop laparoscopy to open surgery. However, if they were operating in the U. S., most would prefer gas laparoscopy to open surgery. **D** Singaporean surgeons (*N* = 5 for this figure only) had mostly similar feedback as that of the U.S. surgeons on ease of using KeyLoop. **E** Singaporean surgeons also preferred gas laparoscopy over KeyLoop with regards to safety, and peritoneal exposure but had somewhat greater preference for KeyLoop when it came to fogging. **F** If surgeons were in an LMIC, they would prefer KeyLoop laparoscopy to open surgery. This was in line with their preference for gas or KeyLoop laparoscopy over open surgery for operating in Singapore

Surgeons from the U.S. and Singapore were asked to complete a survey regarding their experience using KeyLoop and the feasibility of using KeyLoop for various laparoscopic cases. Among the six surgeons from Singapore, three were female while three were male. There were three pediatric surgeons, one laparoscopic surgeon, one surgical oncologist, and one surgical resident. Four surgeons had prior surgical experience in LMICs. Five surgeons routinely performed laparoscopic cases in their routine practice. Figure 6 presents surgeons’ feedback on the ease of using the KeyLoop.

In the U.S., four surgeons found inserting KeyLoop to be very easy or easy. Forty percent of surgeons could very easily adjust KeyLoop while 20% noted difficulty. Forty percent of surgeons could easily achieve sufficient exposure using KeyLoop while others were more neutral in their view. Eighty percent of surgeons found removing KeyLoop easy or very easy and 40% found using the stand very easy with others being neutral (Fig. 6A). In terms of laparoscope fogging, safety, and peritoneal exposure, surgeons preferred to use gas laparoscopy, rather than the KeyLoop (Fig. 6B). If surgeons were in an LMIC, they had varied responses over whether they would prefer KeyLoop laparoscopy to open surgery. However, if they were operating in the United States, most would prefer gas laparoscopy to open surgery (Fig. 6C).

Data regarding ease of use was missing for one Singaporean surgeon, but four surgeons found inserting KeyLoop to be very easy or easy. Sixty percent of surgeons could easily adjust KeyLoop while 40% noted difficulty. Eighty percent of surgeons could easily achieve sufficient exposure using KeyLoop with others were neutral in their view. All surgeons found removing KeyLoop and using the stand easy or very easy (Fig. 6D). For laparoscopic safety and peritoneal exposure, surgeons preferred to use gas laparoscopy over KeyLoop while they prefered Keyloop over gas laparoscopy in the case of fogging (Fig. 6E). If surgeons were in an LMIC, they would prefer KeyLoop laparoscopy over open surgery. If they were operating in Singapore, most would prefer gas laparoscopy to open surgery (Fig. 6F). Both US surgeons with experience in LMICs stated that they would prefer KeyLoop over open surgery in LMICs. Three of the five Singaporean surgeons with experience in LMICs stated that they would prefer KeyLoop over open surgery in LMICs.

**Fig. 7:**
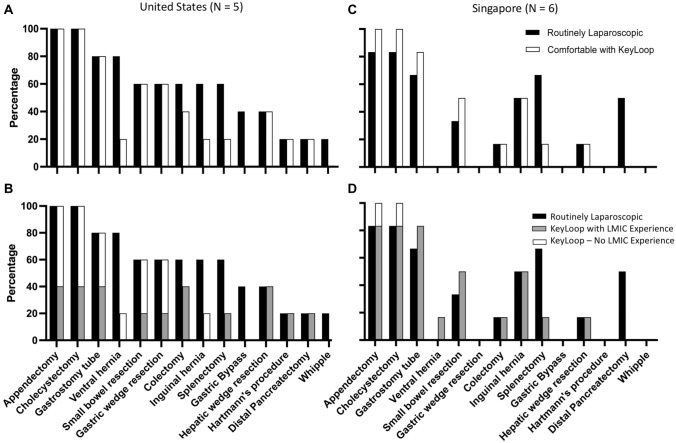
Surgeons’ self-reported routine use of laparoscopy and willingness to use KeyLoop. **A** US surgeons were asked to mark which cases they routinely perform laparoscopically (solid bars). They were then asked to mark which cases they would feel comfortable to perform with KeyLoop laparoscopy (unfilled bars). When U.S. surgeons routinely performed appendectomies, cholecystectomies, gastrostomy tubes, small bowel resections, gastric wedge resections, hepatic wedge resections, Hartmann’s procedures and distal pancreatectomies, then they felt comfortable to perform these procedures with KeyLoop. They felt less comfortable performing ventral hernia repairs, colectomies, inguinal hernia repairs and splenectomies. Surgeons did not feel comfortable performing gastric bypass or Whipple procedures with KeyLoop. **B** Surgeon comfort with using the KeyLoop, separated by those U.S. surgeons with and without experiences operating in LMICs. **C** When Singaporean surgeons routinely performed appendectomies, cholecystectomies, gastrostomy tubes, small bowel resection, inguinal hernia repairs, hepatic wedge resection, and colectomies, then they unanimously felt comfortable to perform these procedures with KeyLoop. They felt less comfortable performing splenectomies. Surgeons did not feel comfortable performing ventral hernia repairs, gastric wedge resection, gastric bypass, distal pancreatectomies, Hartmann’s procedures, or Whipple procedures with the KeyLoop (Fig. 7C). **D** Surgeon comfort with using the KeyLoop, separated by those Singaporean surgeons with and without experiences operating in LMICs

Both U.S. and Singaporean surgeons were asked to select laparoscopic procedures that they routinely perform laparoscopically from a list of surgical procedures (Figs. 7A and C). They were given the same list of procedures and asked to mark which procedures they would feel comfortable to perform with KeyLoop. When U.S. surgeons routinely performed appendectomies, cholecystectomies, gastrostomy tubes, small bowel resections, gastric wedge resections, hepatic wedge resections, Hartmann’s procedures and distal pancreatectomies, then they unanimously felt comfortable to perform these procedures with KeyLoop. They felt less comfortable performing ventral hernia repairs, colectomies, inguinal hernia repairs, and splenectomies. For ventral and inguinal hernia repairs they noted that the KeyLoop may be in the way of the hernia, or it may not provide enough exposure in the lower abdomen/groin. Surgeons did not feel comfortable performing gastric bypass or Whipple procedures with the KeyLoop, arguably due to greater surgical skills complexity involved in these procedures and the larger body habitus of patient’s requiring gastric bypass.

When Singaporean surgeons routinely performed appendectomies, cholecystectomies, gastrostomy tubes, small bowel resection, inguinal hernia repairs, and hepatic wedge resection, and colectomies, then they unanimously felt comfortable to perform these procedures with KeyLoop. They felt less comfortable performing splenectomies. Surgeons did not feel comfortable performing ventral hernia repairs, gastric wedge resection, gastric bypass, distal pancreatectomies, Hartmann’s procedures, or Whipple procedures with the KeyLoop (Fig. 7C).

For both U.S. and Singaporean surgeons, these responses were also separated into surgeons with and without previous experience operating in an LMIC (Figs. 7B and D). The comfort level for using KeyLoop did not vary with their experience in LMICs.

## Discussion

We demonstrate that KeyLoop is easy to use and has comparable operative times, blood loss, risk of complications and tissue damage relative to SOC gas laparoscopy. Surveyed surgeons suggested that they would use KeyLoop for common surgical procedures. They would prefer to use gas laparoscopy in their current clinical practice but would feel comfortable to use KeyLoop in settings where gas is not available.

### Gasless laparoscopy

When laparoscopy was under development in high-income countries (HIC) as an alternative to open surgery, gasless laparoscopy was explored [13, 26]. However, gas insufflation was found to be superior for gaining exposure, and because medical grade carbon dioxide and constant electricity are easily sourced in HICs, gas laparoscopy became the standard of care in line with the needs of the HIC surgeons [26]. However, needs and resource constraints of LMIC surgeons are different requiring frugal innovation [9]. Several instances of abdominal distention systems developed by LMIC surgeons and biomedical engineers have been previously described across case reports and proof-of-concept studies [14–18]. Robust studies on safety and practical utility of well-tested retractor systems for gasless laparoscopy remain limited. To fill the evidence gap, we describe the preclinical findings of KeyLoop testing in a porcine model.

The design approach and findings from bench testing of KeyLoop have been presented previously [21]. Mechanical retractors of various sizes have been optimized through careful anatomical considerations and mathematical modeling to ensure sufficient exposure of the abdominal cavity for gasless lift laparoscopy. KeyLoop has been shown to have good structural integrity, an easy sterilization process, and manufacturing possibility in LMICs. Advantages of KeyLoop along with its potential role in improving access to affordable laparoscopy in LMICs, particularly in sub-Saharan Africa, have been previously reported [19].

### Feasibility and safety of using KeyLoop

Here our goal was to present pre-clinical evidence for feasibility and safety of using KeyLoop for common laparoscopic surgeries. We observed similar operative times and minimal complications for KeyLoop compared to gas laparoscopy. Cholecystectomies were the most difficult among the assessed surgical tasks, and there was some learning effect in adapting to the porcine anatomy. Notably, the operative time of about 7 min for cholecystectomy using KeyLoop in pigs was shorter to that typically observed in humans [27], which is due to the lack of inflammation and straightforward anatomy of Calot’s Triangle.

One surgeon in the study, while rushing to complete the task, did cause a bowel injury during KeyLoop insertion. Of note, the surgeon was rushing through the procedure as they were running late for clinic and did not follow the safety instructions for insertion. Our team reviewed this injury, as well as the design of the KeyLoop. The KeyLoop has a very smooth end, and it was not felt that there was a way to improve this aspect of the design. We plan to use this injury example in the training manual for the KeyLoop. Therefore, when inserting the KeyLoop it is important to proceed with caution and always inspect the bowel for injury. This is also true for gas laparoscopy, as many injuries have been reported with laparoscopic trocar insertion [28]. We also observed that during cautery use with KeyLoop, smoke tended to accumulate in the peritoneal cavity. This was easily improved by applying a low level of negative pressure (suction) to one of the vents on the laparoscopic ports to evacuate the smoke.

While not statistically significant, we found for heart rate and blood pressure that the distribution had a large variance, with higher values above the median in pigs that underwent gas laparoscopy compared to those using KeyLoop. This is likely due to the ventilatory and hemodynamic changes caused by the increased intraperitoneal pressure [29, 30]. Previous studies comparing pneumoperitoneum to gasless laparoscopy found that descending aorta blood flow, central venous pressure and heart rate were higher in the pneumoperitoneum group [31]. Therefore, our results are consistent with previous reports. In a recent study from South Korea, a retrospective review found no differences in complications for gynecologic surgery performed with pneumoperitoneum versus gasless laparoscopy [11]. Hence, gasless laparoscopy can be safely performed, and there is a potential safety benefit in patients with cardiovascular compromise.

We surveyed surgeons in the U.S., and Singapore. While there are some minor differences such as differences in routinely performed laparoscopic procedures and the greater degree of preference for using KeyLoop in an LMIC compared to open surgery among Singaporean surgeons, the surgeons in both locations shared similar views on major issues. The surgeons found that it was easy to mount, insert, and use the KeyLoop. While they preferred gas insufflation over KeyLoop, they were able to perform a variety of operative tasks using KeyLoop. They did not feel comfortable to use the KeyLoop for complex surgeries such as a Whipple procedure or surgeries in obese patients such as gastric bypass. It was also felt that for ventral hernias, the KeyLoop device may pose a physical barrier to mesh placement and for inguinal hernias it may not provide enough exposure in the inguinal region, although this procedure was not tested in this study. Nevertheless, surgeons felt that KeyLoop can be used safely for many basic surgical procedures. Similarities in feedback from surgeons practicing in two different countries substantively support feasibility of using KeyLoop in different practice settings. A previous survey of COSECSA member surgeons has shown that African surgeons are very eager to increase their use of laparoscopy, and are interested in gasless laparoscopy [9]. Hence, we are not proposing that KeyLoop will replace gas laparoscopy as the standard of care, but rather will provide a means for laparoscopic surgery to be performed in areas where gas insufflation is not accessible.

### Training and scale-up of laparoscopy in LMICs

While the exposure attained using KeyLoop may not be preferred over gas insufflation, it is useful for conducting safe laparoscopies in settings that lack gas insufflation capacity. KeyLoop should not be considered a replacement for gas laparoscopy. Rather, it can be used as an entry point for training LMIC surgeons in laparoscopic surgical techniques and scaling up laparoscopy in settings where operating theaters lack gas insufflation. Introduction of KeyLoop in LMICs, especially those in sub-Saharan Africa is timely. Under initiatives run by the College of Surgeons of East, Central, and Southern Africa (COSECSA), West African College of Surgeons (WACS) and Pan African Association of Christian Surgeons (PAACS) training of surgeons and their retention in the region has expanded several fold in the past decades [32–35]. While some training sites do provide opportunities for training in laparoscopic surgery, increasing integration of laparoscopic training in these programs could help create a generation of surgeons who will further the scale up of minimally invasive surgeries in the region. KeyLoop can be used for educating the current surgical trainees who can then mentor others in the future.

There has been a rise in efforts to train surgeons in laparoscopic skills across LMICs. For instance, WACS conducts workshops on Basic Laparoscopic and Endoscopic Surgical Training (BLEST) [35]. Fundamentals of Laparoscopic Skills (FLS) in low-resource settings has also shown to be feasible in Botswana [36]. Efforts using virtual platforms for laparoscopic training have become popular in other LMICs under the Global Laparoscopic Program run by the Society of American Gastrointestinal and Endoscopic Surgeons [37]. More innovative approaches such as tele-simulation, where surgical simulators across two locations are linked by the internet for surgeons located elsewhere to be able to train surgical trainees in LMICs, have also been attempted [38]. However, continuation and scaling up of these efforts require safe, economical and easy-to-use surgical equipment. Integrating KeyLoop into existing training curriculums can help scale up laparoscopic training and provide a sustainable method for surgeons to practice laparoscopy in their local operating rooms. Multiple studies have noted that lack of equipment, due to high costs and maintenance, is one of the most important barriers to laparoscopic training and practice [6, 39]. Within LMICs, high-cost equipment can be made available through large international donors only in select urban and tertiary hospitals, thereby furthering inequities for patients and surgical trainees. Sustainable partnerships that rely on cost-effective equipment designed to meet the contextual needs, such as KeyLoop, are needed to ensure equitable laparoscopic training of LMIC surgeons in the future [19, 40].

### Limitations

Pigs have relatively thin abdominal walls, and therefore the upward lifting force may be greater in humans, particularly obese patients. This may impact the ease of use and exposure that can be obtained. A future First-In-Human study will be performed to assess the feasibility of using KeyLoop in a clinical setting. However, it is promising that there are case reports of other centers successfully using lift laparoscopy to perform basic surgeries. Secondly, the success of laparoscopy is not merely determined by the operative tools available, but also dependent on the skills of the operating surgeon. The surgeons in this study were highly skilled in laparoscopy, having all received dedicated fellowship training and routinely perform complex laparoscopic cases in their clinical practice. It will be imperative in a scale-up model of laparoscopy using KeyLoop that surgeons receive adequate training and mentorship through laparoscopic courses and capacity building efforts.

This study was performed in HICs, as the logistics of performing the study with our partners in Uganda was not feasible at this time due to limited numbers of laparoscopically-trained surgeons who are clinically overloaded with patient care, and the fact that there is very limited laparoscopic equipment, all of which is reserved for patient use. There is not an animal vivarium with laparoscopic equipment at this time. However, we acknowledge that operative feedback from our African colleagues will be valuable, and we have a subsequent study planned in which they will be the lead surgeons in a first-in-human clinical trial.

Some surgeons found that KeyLoop was difficult to adjust, and this is a limitation of this technology. It is a self-retaining retractor that must be positioned before starting the case. It provides very stable and strong exposure, but the trade-off is that it takes some work to reposition it in the middle of the case. This is similarly true for other self-retaining retractors and laparoscopic liver retractors. We are currently modifying the stand to make the tightening of the joints more user friendly.

The feedback survey contained hypothetical and subjective questions, designed to assess perceived feasibility. Concerning performance of specific surgical procedures, there may be differences between what surgeons *feel* they can do, and what they actually *can* do. Future studies will be needed to assess the clinical feasibility.

## Conclusions

KeyLoop is a self-retaining retractor, used to provide peritoneal exposure during gasless laparoscopy. In this porcine model, KeyLoop was compared to standard of care gas laparoscopy and found to have similar operative times, hemodynamic stability, ease of use, and minimal complications. KeyLoop was not associated with any histologic damage to the abdominal wall. Surgeons who routinely perform laparoscopic surgery in the U.S. and Singapore felt that KeyLoop can be used to safely perform a variety of common laparoscopic surgeries. KeyLoop will not replace gas laparoscopy as the standard of care but can provide a feasible alternative for surgeons in LMICs lacking access to gas laparoscopy.

## Supplementary Information

Below is the link to the electronic supplementary material.

Supplementary file1 (PDF 425 kb)

## Abbreviations

SOC: Standard of care
LMIC: Low- and middle-income country
SSA: Sub-Saharan Africa
