# Supplementary material for: KeyLoop retractor for global gasless laparoscopy: evaluation of safety and feasibility in a porcine model
Source: Surg Endosc. 2023 Apr 19;37(8):5943–55. doi: 10.1007/s00464-023-10054-5 (PMC10338623; doi:10.1007/s00464-023-10054-5)
Supplement: Supplementary file 1 — Supplementary file1 (PDF 425 kb) [file 464_2023_10054_MOESM1_ESM.pdf]

My First Instrument

Please complete the survey below.

Thank you!

We have developed prototypes for a laparoscope and lift retractor with the goal of making laparoscopic surgery more affordable and accessible in places where it is not currently available, mainly low- and middle-income countries. The laparoscope (KeyScope) is low-cost (~$120), connects to a laptop via a USB cord for viewing images and powering the device, and is a single component that can be cleaned via submersion in chemical disinfectants. The retractor (KeyLoop) mechanically lifts the abdominal wall and does not use power or CO2 for insufflation.

We wish to obtain feedback from surgeons regarding the prototypes, so that we can make changes to them, if needed, before moving to a clinical study. The benefit of completing the survey is to help us improve the prototypes and continue developing affordable, accessible and relevant laparoscopic equipment. There are no known risks associated with completing the survey.

Completion of the survey indicates implied consent to participate. If you wish to decline, simply do not fill out the survey. It should take about 15 minutes to complete, is voluntary and anonymous. The study team will not have access to any personal information about you.

---

1) Have you ever performed surgery in a low- and middle-income country before?

◯ Yes
◯ No

---

2) Please check the procedures that you routinely perform LAPAROSCOPICALLY as part of your surgical practice:

☐ Appendectomy
☐ Gastrostomy or Jejunostomy tube
☐ Inguinal hernia
☐ Cholecystectomy
☐ Ventral hernia
☐ Small bowel resection
☐ Gastric wedge resection
☐ Colectomy with primary anastomosis
☐ Hartmann's procedure
☐ Splenectomy
☐ Roux-en-Y Gastric Bypass
☐ Hepatic wedge resection
☐ Distal Pancreatectomy
☐ Whipple

---

3) Please check all of the procedures you feel that you could perform with the KeyLoop.

☐ Appendectomy
☐ Gastrostomy of Jejunostomy Tube
☐ Inguinal Hernia
☐ Cholecystectomy
☐ Ventral Hernia
☐ Small Bowel Resection
☐ Gastric Wedge Resection
☐ Colectomy with Primary Anastomosis
☐ Hartmann's Procedure
☐ Splenectomy
☐ Roux-en-Y Gastric Bypass
☐ Hepatic Wedge Resection
☐ Distal Pancreatectomy
☐ Whipple

4) How would you rate the exposure obtained from KeyLoop compared to Standard of Care?

1: Standard of Care was much better | 50: They were about the same | 100: KeyLoop was much better

(Place a mark on the scale above)

5) Once you obtained exposure, were you able to maintain the exposure during the whole case?

◯ Yes, definitely
◯ Mostly, the exposure was adequate
◯ Not completely, but good enough to complete the case
◯ Maintaining exposure was difficult
◯ No, unable to complete case

How easy was it to...

		Very difficult	Difficult	Neutral	Easy	Very Easy
6)	insert the KeyLoop?	◯	◯	◯	◯	◯
7)	adjust the KeyLoop?	◯	◯	◯	◯	◯
8)	achieve sufficient exposure?	◯	◯	◯	◯	◯
9)	remove the KeyLoop?	◯	◯	◯	◯	◯
10)	use the stand?	◯	◯	◯	◯	◯

11) How was the height of the stand holding up the KeyLoop?

◯ Too low
◯ Slightly low
◯ Just at the perfect height
◯ Slightly high
◯ Too high

12) How did you feel about the KeyLoop stand?

◯ It was in the way, and I could NOT work around it.
◯ It was in the way, but I could work around it.
◯ It was a little awkward, but did not affect the procedure
◯ It was not in the way.

13) How would you rate the overall safety of the KeyLoop Retractor?

1: Using Standard of Care is more safe | 50: They seemed equally safe | 100: Using KeyLoop is more safe

(Place a mark on the scale above)

14) Please rank the fogging of the laparoscope when using KeyLoop compared to standard of care:

1: Much less fogging with gas | 50: No difference in fogging using gas or KeyLoop | 100: Much less fogging with KeyLoop

(Place a mark on the scale above)

15) In your current practice, do you prefer to perform open or laparoscopic surgeries?

1: Strongly prefer open surgery | 50: No preference between open and laparoscopic surgery | 100: Strongly prefer laparoscopic surgery

(Place a mark on the scale above)

16) If you were operating in a low- or middle-income country and gas laparoscopy was not available, what way would you prefer to do the case?

1: Strongly prefer open surgery	50: No preference between open surgery and KeyLoop	100: Strongly prefer using KeyLoop

(Place a mark on the scale above)

17) Tell us about any problems you experienced while using the KeyLoop, or any feedback / suggestions.

18) Tell us what you liked about the KeyLoop.
